# Validity and reliability of transbronchial needle aspiration for diagnosing mediastinal adenopathies

**DOI:** 10.1186/1471-2466-10-24

**Published:** 2010-04-28

**Authors:** Alberto Fernández-Villar, Maribel Botana, Virginia Leiro, Ana González, Cristina Represas, Alberto Ruano-Raviña

**Affiliations:** 1 Bronchoscopy Unit, Pulmonary Department, Xeral-Cíes Hospital, University Hospitalary Complex of Vigo, C/Pizarro 22; 36204 Vigo, Spain; 2 Patology Department, Xeral-Cíes Hospital, University Hospitalary Complex of Vigo, Spain; 3 Galician Agency for Health Technology Assessment, Department of Health, Galician Regional Authority, Spain. Edif. Admtvo San Lázaro s/n. 15781 Santiago de Compostela Spain; 4 Department of Preventive Medicine and Public Health, University of Santiago de Compostela, Spain

## Abstract

**Background:**

The aim is to assess the validity and reliability of transbronchial needle aspiration (TBNA) of mediastinal and hilar adenopathies and to evaluate factors predictive of TBNA outcome.

**Methods:**

We performed an analysis of prospectively collected data of patients (n = 580) who underwent TBNA (n = 685) from January 1998 to December 2007 in our center. Validity and reliability were evaluated for the overall sample and according to specific pathology. Factors predicting the successful acquisition of diagnostic samples were analyzed by multivariate analysis.

**Results:**

Overall sensitivity, specificity, accuracy, and positive and negative predictive (NPV) values for TBNA were 68%, 100%, 68.8%, 100%, and 10%, respectively. The most sensitive and accurate TBNAs were obtained for patients with small cell lung carcinoma and the worst results were for patients with lymphomas. NPV were similar for all pathologies. The most predictive factors of outcome were adenopathy size and the presence of indirect signs at the puncture site.

**Conclusion:**

The sensitivity and accuracy of TBNA are high in small cell lung cancer, followed by other types of carcinoma, sarcoidosis, and tuberculosis, and low for lymphoproliferative diseases. The NPV of TBNA for all individual pathologies is low. The size of the adenopathy and the presence of indirect signs at the puncture site predict the achievement of diagnostic samples.

## Background

Transbronchial needle aspiration (TBNA) is a minimally invasive bronchoscopic technique that is cost-effective and safe for diagnosing mediastinal and hilar adenopathies in patients with lung cancer and other pathologies, including other cancers, sarcoidosis, and infectious processes such as tuberculosis [1,2]. However, there is great variability in the results obtained depending on endoscopist experience [3], adenopathy characteristics and their location [4-6], type of needle used [1,2,7], etiology of mediastinal adenopathies [8], possibility of an immediate evaluation by a cytopathologist [9], or use of accessory techniques such as bronchoscopic ultrasound for adenopathy detection [10]. Further, factors that can influence the evaluation of the outcome are prevalence of underlying pathologies [11] and interpretation of the results from other published studies [12,13]. Most of the studies on the validity and reliability of TBNA as diagnostic tool for mediastinal and hilar adenopathies have been performed in patients with lung cancer. Few studies have analyzed the sensitivity, specificity, predictive values, and TBNA accuracy for diagnosing diseases that produce frequently mediastinal adenopathies [8] and those that have small sample sizes. Therefore, it is difficult to compare the results among studies and perform a global evaluation of the technique.

The purpose of this study was to evaluate the validity and the predicitive value of TBNA for diagnosing mediastinal adenopathies of different etiologies in a large cohort of patients treated at a tertiary care hospital over 10 years and to determine which factors might predict TBNA outcome.

## Methods

### Study Design and Scope

We performed an analysis of prospective collected data of patients with thoracic computerized tomography (CT)-detected mediastinal and hilar adenopathies adjacent to the tracheobronchial tree (with the short axis ≥ 10 mm) who underwent consecutively TBNA at Xeral Hospital of Vigo from January 1998 to December 2007. Xeral Hospital of Vigo is a tertiary care hospital which is part of the Vigo University Hospitalary Complex and serves 250,000 people. About five hundred bronchoscopies are performed annually. TBNAs were performed by experienced bronchoscopists and residents under their supervision. Those patients for whom a final diagnosis could not be obtained were excluded from the study. Part of these patients were included in some previous studies [6,14,15].

### TBNA Materials and Methods

Different types of cytology needles [MW-122 (22 gauge; Bard-Wang, Billerica, USA), NA-401D-1321 and NA-401D-1321 (21 gauge; Olympus, Tokyo, Japan), and eXcelon 21 gauge (Boston Scientific, Boston, USA)] and a unique model of histology needle [MW-319 (19 gauge; Bard-Wang, Billerica, USA)] were used to perform TBNA. Needle type selection depended on availability at the time of TBNA and on the clinical suspicion of patient's pathology. Histology needles were used when the pathology was suspected to be of benign origin or lymphoproliferative processes; cytology needles were used in all the other cases.

Patient preparation for TBNA was the same as for all diagnostic bronchoscopies performed in the hospital. After the procedure, the patient's personal data, age, sex, description and location of the lesions detected in thoracic CT, endoscopic findings including the presence of indirect signs such as hyperemia, edema, or extrinsic compression of the carina or widening at the puncture point, were recorded on a standardized form. Finally, all patients were followed until the moment a definitive diagnosis was made.

Potential risks of the endoscopic techniques were explained to all patients and all patients provided informed consent. Patients were pre-medicated with 0.5 mg atropine intramuscularly and underwent conscious sedation with intravenous midazolam. Bronchoscopy was performed transnasally using 2% lidocaine as local anesthesia with the patient in the supine decubitus position. TBNA of selected mediastinal or hilar adenopathies stations was performed before bronchial tree exploration, avoiding bronchoscopic aspiration or contamination with secretions, as far as possible. The insertion point was determined after a careful analysis of thoracic CT and following previous recommendations by other authors [1,2]. When several adenopathies were selected, TBNA was carried out at the most accessible and largest adenopathy. We collected a sample from another station only if we could not obtain at least one adequate sample from the primary station after three or four attempts. A cytopathologist was present during the procedure and made an immediate microscopic evaluation of the cytology samples and a macroscopic evaluation of histology samples. Cases with a lesion on the tracheobronchial mucosa at the puncture point were not included in the study.

### Data interpretation

All samples with high lymphoid cellularity (at least 30% [5]) suggesting a lymph node puncture or the existence of many neoplastic cells or cytological/histological findings that allowed for a specific diagnosis ("diagnostic samples") were considered "adequate samples". Samples with atypias or dubious, mucousy, bloody, or tracheobronchial wall cellularity were considered "non-adequate".

In all patients with TBNA performed in more than one lymph node station, the best diagnosis or the most adequate, if there was disagreement, was selected. Every patient was considered as an analysis unit [16].

All diagnostic samples, those with abundant lymphoid cellularity confirmed by a surgical technique, and those with benign pathology that did not change or disappeared after six months were considered true positives (TP). TPs were not further confirmed by a surgical technique due to the very high specificity of TBNA; false positives (FP) are extremely infrequent. Cases yielding only a very high lymphoid cellularity in patients with specific pathologies such as carcinomas, lymphomas, tuberculosis, or sarcoidosis, and whose surgical biopsies produced results similar to those of TBNA were considered true negatives (TN). The samples with lymphoid cellularity not confirmed by surgical techniques or follow-up and all patients with non-adequate TBNAs were considered false negatives (FNs).

The diagnosis of carcinoma or lymphoma was made according to standard criteria. Sarcoidosis was diagnosed when sarcoid granulomas were found and other diseases with similar clinical or radiologic findings could be reasonably excluded. Tuberculosis was diagnosed when the cytohistologic samples showed necrotizing granulomas with or without *Mycobacterium tuberculosis *isolation and the patient had a good response to antituberculosis treatment. Anthracotic or reactive adenopathies were diagnosed according to clinical criteria, occupational exposure, cytohistologic results (numerous benign lymphocytes and resolution of patient process or few lymphocytes plus numerous anthracotic pigment-laden macrophages), and clinical-radiologic follow-up (stabilization or disappearance of the nodes), and were considered non-specific pathologies.

### Statistical analysis

Qualitative variables are reported as absolute frequencies and percentages and numeric variables are reported as median and range [median (range)]. Sensitivity, specificity, positive predictive value (PPV), negative predictive value (NPV) and accuracy were determined using the standard definitions.

Comparison of discrete variables was performed by Chi squared or the Fisher exact test. All factors associated with the acquisition of diagnostic samples with a p-value < 0.20 in univariate analysis were used in multivariate analysis; odds ratios (OR) and 95% confidence intervals (95% CI) were determined. In the logistic regression model, the quantitative variables were divided in two groups: lesser than and greater than or equal to the median, in order to obtain more potent estimations. Underlying pathologies were classified into two groups: malignant (carcinomas or lymphomas) and benign (all other causes). Analyses were performed with SPSS, version 15.0 (SPSS, Chicago, IL, USA).

In all cases a signed informed consent form was obtained. The study protocol was approved by the Ethics Committee of the Xeral Hospital (Vigo, Spain).

## Results

### Population description

Five hundred and eighty patients were included in the study. Six hundred and eighty-five TBNAs were performed in different lymph node stations (479 in one site only, 97 in two sites, and four in three sites). The age of the patients was 62.0 (21-89) years, 422 (72.8%) males. Cytology needles were used in 414 (71.4%) cases and histology needles were used in 166 (28.6%) cases. Patient characteristics and final diagnoses for all patients are presented in Table 1.

**Table 1:** Final diagnoses, patient characteristics, needle type used, and cases in which TBNA was the only technique that permitted the diagnosis.

Pathology	Patients, n (%)	**Age**^# ^**in years**	Male, n (%)	Cytology needle, n (%)	Unique diagnostic, n (%)
Non-small cell lung carcinoma^¶^	280 (48.3%)	62.0 (42-87)	221 (79.5%)	262 (93.6%)	96 (34.2%)
Small cell lung carcinoma	74 (12.8%)	65.0 (34-89)	62 (83.8%)	62 (83.8%)	30 (40.5%)
Extrapulmonary carcinoma^+^	44 (7.6%)	61.5 (26-81)	21 (47.7%)	34 (77.3%)	16 (36.3%)
Sarcoidosis	57 (9.8%)	39.5. (21-75)	29 (58.9%)	4 (7.0%)	20 (35.1%)
Tuberculosis	19 (3.3%)	58.0 (38-79)	11 (57.9%)	3 (15.8%)	8 (42.1%)
Lymphoma	15 (2.6%)	66.0 (29-81)	7 (46.7%)	3 (20.0%)	4 (26.7%)
Anthracotic and reactive	91 (15.7%)	63.0 (21-85)	71 (78.0%)	46 (50.5%)	11 (12.1%)
Total	580	62.0 (21-89)	422 (72.8%)	414 (71.4%)	214 (31.8%)

^#^Data are presented as median (range).

^¶^130 adenocarcinomas, 83 large-cell undifferentiated carcinomas, 67 squamous cell carcinomas.

^+^13 breast, 10 renal, 5 larynx, 4 colon, 3 gastric, 2 hepatocarcinoma, 2 prostate, 1 endometrium, 1 ovary, 1 bladder, 1 thymus, 1 esophagus.

The smallest axis diameter of the studied adenopathies was 20.0 (10-85) mm; 274 (47.2%) were smaller than 20 mm. An median of 2.0 (1-6) passes were performed. The frequencies at which TBNA was performed for specific nodes were right paratracheals, 320; subcarinals, 238; left paratracheals, 62; hilars, 30; principal bronchus, 5; precarinals, 28; and retrocarinals, 2. Table 2 shows the characteristics of the adenopathies detected for the sample as a whole and according to patient illness.

**Table 2:** Characteristics of the mediastinal and hilar adenopathies.

Pathology	Lymph nodes examined	Lymph nodes per patient^#^	Passes^#^	Lymph node size, mm^#^
Non-small cell lung carcinoma	314	1.0 (1-2)	2.0 (1-5)	19.0 (10-85)
Small cell lung carcinoma	78	1.0 (1-2)	2.0 (1-6)	28.5 (10-79)
Extrapulmonary carcinoma	55	1.0 (1-3)	2.0 (1-6)	21.0 (10-42)
Sarcoidosis	82	1.0 (1-3)	3.0 (1-4)	20.0 (10-45)
Tuberculosis	27	1.0 (1-2)	3.0 (2-4)	20.0 (10-39)
Lymphoma	20	1.0 (1-2)	3.0 (2-5)	23.0 (10-41)
Anthracotic and reactive	109	1.0 (1-3)	3.0 (1-6)	15.0 (10-59)
Total	685	1.0 (1-3)	2.0 (1-6)	20.0 (10-85)

^#^Data are presented as median (range).

### TBNA Validity and Reliability

Adequate samples were obtained for 439 (75.7%) patients. Cytohistologic diagnoses could be made for 343 (59.1%) patients and there was abundant lymphoid cellularity in 96 (16.6%) patients. Samples obtained through TBNA were considered non-adequate for 141 (24.3%) patients. Of the 237 patients for whom a specific diagnosis could not be made based on TBNA (141 with inadequate samples + 96 with only lymphoid cellularity in the samples), diagnoses were ultimately established for 106 (44.7%): 65 patients were diagnosed using surgical techniques and 41 patients were diagnosed during clinical-radiologic follow-up.

Table 3 shows the sensitivity, specificity, PPV, NPV and accuracy for TBNA. Sensitivity and accuracy were lower for lymphomas than for other pathologies except anthracotic and reactive adenopathies. The NPV was low for every diagnosis group and there were no significant differences among the groups.

**Table 3:** Validity, reliability, and pre-test probability of TBNA outcome for diagnosing mediastinal and hilar adenopathies.

Pathology	Patients	Se	Sp	PPV	NPV***	Accuracy
Non-small cell lung carcinoma*	280	68.3%	100%	100%	9.5%	69.3%
Small cell lung carcinoma**	74	93.2%	100%	100%	16.7%	93.2%
Extrapulmonary carcinoma*	44	69.8%	100%	100%	7.1%	70.5%
Sarcoidosis*	57	63.6%	100%	100%	9.1%	64.9%
Tuberculosis*	19	72.2%	100%	100%	16.7%	73.7%
Lymphoma	15	35.7%	100%	100%	10.0%	40.0%
Anthracotic and reactive	91	52.7%	-	100%	-	52.7%
Total	580	68.0%	100%	100%	10.0%	68.8%

Se, sensitivity; Sp, specificity; PPV, positive predictive value; NPV, negative predictive value.

* p of sensitivity and accuracy < 0.05 compared with the lymphoma group.

** p of sensitivity and accuracy < 0.001 compared with all other pathologies.

*** No statistically significant differences between the various pathologies.

The diagnoses of 63 of the patients whose samples had abundant lymphoid cellularity and could not support a specific diagnosis were ultimately verified. Of them, 42 were considered TPs (all were anthracotic or reactive adenopathies), 14 were TNs, and seven were FNs (sensitivity, 85.7%; NPV, 66.7%; accuracy, 88.9%).

### Factors Influencing the Acquisition of Diagnostic Samples

In univariate analysis (Table 4), factors associated with the acquisition of diagnostic samples with p < 0.20 were type of disease, type of needle used, adenopathy diameter, and the existence of indirect signs at the insertion site. In multivariate analysis, only adenopathy diameter and the existence of indirect signs at the insertion site were independent predictors of acquiring a diagnostic sample (Table 4).

**Table 4:** Univariate and multivariate analysis of factors predicting the acquisition of diagnostic samples for mediastinal and hilar adenopathies by TBNA.

Factors	Diagnostic samples	Univariate analysis	Multivariate analysis	
		p-value	OR (95% CI)	p-value
Sex				
Male	291/422 (69.0%)	0.9	-	-
Female	106/154 (68.8%)			
Age				
< 60 years	191/275 (69.5%)	0.7	-	-
≥ 60 years	202/297 (68.0%)			
Type of disease				
Malignant	297/413 (71.9%)	0.07	1.2 (0.7-2.0)	0.3
Benign	101/167 (60.5%)			
Type of needle				
Cytology	291/414 (70.3%)	0.1	1.1 (0.7-1.9)	0.4
Histology	107/166 (64.5%)			
Lymph node station				
Right paratracheal	161/234 (68.8%)	0.3	-	-
Subcarinal	106/147 (72.1%)			
Left paratracheal	36/50 (72.0%)			
Others	34/47 (72.3%)			
Combination (2 or more)	61/102 (60.0%)			
Lymph node size				
< 20 mm	159/274 (58.0%)	0.0001	2.2 (1.4-3.2)	0.0001
≥ 20 mm	239/306 (78.1%)			
Indirect sign				
No	246/391 (62.9%)	0.0001	1.7 (1.1-2.6)	0.02
Yes	152/189 (80.4%)			

CI: confidence interval

### Complications

Complications related to TBNA occurred in 12 patients (2.1%): 11 cases of mild to moderate, self-limited bleeding at the puncture site and one case of pneumomediastinum and subcutaneous emphysema.

## Discussion

The majority of primary studies and even some meta-analyses about the validity and reliability of TBNA in mediastinal adenopathies have been performed in patients with a single type of pathology, mainly non-small cell lung cancer [4-7,11]. Only a few of these studies have evaluated the effectiveness of TBNA for large samples of different pathologies [8]; these studies provide us a broader understanding of the technique's utility.

The validity of a diagnostic tool is the degree to which the test measures what is intended to be measured and is normally determined by evaluating its sensitivity and specificity. The reliability of a diagnostic tool is indicated by the predictive value of a positive or negative result and indicates the reliability with which this test predicts the presence or absence of illness. This probability is influenced by the prevalence of the pathology studied and by the "gold standard" diagnostic techniques used for comparison. The same apply to TBNA, as shown by Holty et al [11] their meta-analysis.

In general, when TBNA is performed, three different situations occur: 1) enough sample is obtained to make a specific cytologic or histologic diagnosis; 2) inadequate or insufficient sample is obtained; or 3) enough sample is obtained for cytology or biopsy (abundant lymphoid cellularity) but it is impossible to affirm whether is the sample is representative of the pathology suspected [17]. In most studies, samples that provided a specific diagnosis were considered TPs, given the high PPV ascribed to the technique [4,5,11,17]. However, there is substantial disagreement about the definitions of FN and TN in the literature; in some studies, only adequate samples were included in the analysis [18,19] which resulted in overestimation of the validity and reliability of TBNA. In other studies, non-adequate samples that were verified but did not have lymphoid cellularity were considered TNs and not FNs. In still other studies, the definition of FN was based on the influence that the TBNA result had in the final decision about patient management [7]. In 15-25% of TBNAs, a representative sample is obtained but a specific diagnosis cannot be made [6,17]. For example, if the patient has a carcinoma, it is possible to find a TN (normal or reactive node) or a FN (metastatic adenopathy, although not appearing in cytologic smears or in biopsies obtained by TBNA). In one recent study, the frequency of such cases was 24% [17]. In our opinion, when the sample is inadequate (a few lymphocytes, mucus, blood, bronchial wall cells, few atypic cells, or not enough tissue sample), the sample should be considered a FN because the cellularity does not represent that of the node. Nevertheless, some authors consider that inadequate samples should not be included when evaluating TBNA validity and reliability as a diagnostic tool [18,19]. TN designations should be reserved for patients with specific pathologies for whom TBNA results were representative of a lymphatic node puncture and the diagnosis was confirmed by other techniques [17,20].

In our study, we found that the validity of TBNA in different pathologies responsible for mediastinal adenopathy varied considerably. The greatest sensitivity and accuracy were obtained for small cell lung carcinoma and the least for lymphoproliferative diseases. This could be due to the higher diameter of adenopathies in patients with small cell carcinoma, as this type of neoplasia is aggressive and its cells have poor adhesion qualities [4,6,8]. The poor results obtained in cases of lymphoma could reflect the need for larger samples to make a specific diagnosis of the type of lymphoma [20]. Moreover, we considered samples that were ambiguous or suggestive but not completely conclusive to be non-adequate. There were no significant differences in TBNA outcomes among non small cell lung carcinomas, extrapulmonary carcinomas, sarcoidosis, and tuberculosis. Anthracotic and reactive adenopathies presented special situations. In many of them, the next step was the follow-up, taking into account the low clinical suspicious of specific processes. However, according to a recently published systematic review [21], these differences are not observed in most published studies when TBNA is performed with ultrasound (EBUS-TBNA) and the sensitivity of EBUS-TBNA is much higher than the reported in this study with convencional TBNA alone.

The low NPV we found for all pathologies (≈ 10%) as compared to published NPVs [18-20] is particularly notable because the NPV may influence patient management. It is possible that the restrictive definitions we used for FN and TN contributed to this low NPV. A recent retrospective study analyzed the results of TBNA of mediastinal adenopathies in 194 patients (157 with lung cancer). The NPV of TBNA was 29% if all results were included and 64% if only adequate samples were evaluated [18]. However, results similar to ours (NPV = 11-20%) were reported in several recent studies using endoscopic ultrasound in patients with lung cancer and sarcoidoisis [10,21-24]. This suggests that any negative test should be verified by other techniques [19,25], even when only a large number of lymphocytes are present, because the NPVs for these cases in our study were less than 70%.

In our study, the factors with the greatest influence on the acquisition of diagnostic samples were the size of the adenopathy and the presence of indirect signs at the puncture point. Other factors such as pathology responsible for the mediastinal adenopahies and type of needle used were not significant. In a study of 166 patients, Sharafkhaneh et al. [8] found that the type of illness and diameter of the node were the most important predictors of TBNA outcome. The differences between their results and ours could be due to the fact that Sharafkhaneh's group obtained lower yields from patients with benign pathologies than malignant pathologies (37% vs. 69%, respectively) [8]. Even though we obtained high sensitivity and accuracy associated with small cell lung carcinoma when all neoplastic processes (except lymphomas) were considered, the type of illness was not an independent predictor of outcome.

In various recent studies, obtaining samples for histology and cytology using needles with the capacity to obtain both types of samples increased the diagnostic yield for different types of pathologies [7,17,26]; however, we found that the type of needle used did not influence the outcome. This might be because we used histology needles for patients suspected of having benign pathology or lymphoproliferative disease. In these cases, cytology samples are not routinely processed.

Our study does have some limitations, most of which are similar to those of other studies concerning the efficacy of TBNA. The most important is the lack of result verification by a surgical "gold standard" technique ([2,19]. However, in light of the high PPV reported for TBNA and that we have considered the worst-case scenarios by classifying all negative TBNAs that were not subsequently confirmed as FNs, this limitation might be less important than in other studies [12]. As well, our study has some advantages over other similar studies, one of which is that the study was conducted by the same team and included a variety of pathologies in a large cohort of patients obtained using a consecutive sampling of all patients who underwent during a 10 year follow-up. Ours represents one of the largest series published to date and the only one that analyzes TBNA validity and reliability as diagnostic tool for differentiating among pathologies that produce mediastinal adenopathies. It should be highlighted that EBUS was not available in most centers when our recruitment period started,

## Conclusion

TBNA is a sensitive and accurate technique for obtaining diagnostic samples from patients with mediastinal adenopathies, especially small cell lung cancer. The size of the adenopathy was the most important predictor of the outcome. For other neoplastic or granulomatous illnesses, although the efficacy of the technique is lower than that observed for small cell lung cancer, its low cost and few complications justify the continued use of classical TBNA as the best initial diagnostic test, especially when other techniques, such as ultrasound bronchoscopy, are unavailable. The reliability of TBNA for mediastinal adenopathies, as determined by its predictive values, was similar for all the analyzed pathologies.

## List of Abreviations

CI: confidence interval; CT: computerized tomography; FN: false negative; FP: false positive; NPV: negative predictive value; OR: odds ratio; PPV: positive predictive value; SD: standard deviation; Se: sensitivity; Sp: specificity; TBNA: transbronchial needle aspiration; TN: true negative; TP: true positive.

## Competing interests

The authors declare that they have no competing interests.

## Authors' contributions

AFV designed the study. AFV, MBR, VLF and CRR performed the bronchoscopys and registered the information provided, implemented the information from the pathological department, performed the statistical analysis, and wrote the manuscript. AGP provided all the information from the pathological department, and reviewed the manuscript thoroughly. ARR helped to interpret the data and with writing. All authors revised critically the manuscript and made intellectual contributions to it.

## Pre-publication history

The pre-publication history for this paper can be accessed here:

http://www.biomedcentral.com/1471-2466/10/24/prepub

## References

[B1] Dasgupta A Mehta AC Transbronchial needle aspiration. An underused diagnostic technique Clin Chest Med 1999 20 39 51 10.1016/S0272-5231(05)70125-8 10205716

[B2] Wang KP Metha A Turner JF Wang KP, Metha A, Turner JF Transbronchial needle aspiration for cytology and histology specimens Flexible Bronchoscopy 2004 2a Cambridge: Blackwell Publisshing 117 137

[B3] Rodríguez de Castro F Díaz López F Juliá Serdá G Rey López A Freixenet Gilart J Cabrera Navarro P Relevance of training in transbronchial fine-needle aspiration technique Chest 1997 111 103 105 10.1378/chest.111.1.103 8996001

[B4] Harrow EM Abi-Saleh W Blum J The utility of transbronchial needle aspiration in the staging of bronchogenic carcinoma Am J Respir Crit Care Med 2000 161 601 607 10673206 10.1164/ajrccm.161.2.9902040

[B5] Patelli M Lazzari L Poletti V Role of fiberscopic transbronchial aspiration in the stating of N2 disease due to non-small cell lung cancer Ann Thorac Surg 2003 73 407 411 10.1016/S0003-4975(01)03447-6 11845850

[B6] Fernández-Villar A Iglesias F Mosteiro M Transbronchial needle aspiration of diseased mediastinal lymph nodes: predictors of positive findings Arch Bronconeumol 2005 41 434 438 16117949 10.1016/s1579-2129(06)60259-0

[B7] Stratakos G Porfyridis I Papas V Exclusive diagnostic contribution of the histology specimens obtained by 19-gauge transbronchial aspiration needle in suspected malignant intrathoracic lymphadenopathy Chest 2008 133 131 136 10.1378/chest.07-1920 17951614

[B8] Sharafkhanch A Baaklini W Gorin A Green L Yield of transbronchial needle aspiration in diagnosis of mediastinal lesions Chest 2003 124 2131 2135 10.1378/chest.124.6.2131 14665491

[B9] Baram D Garcia RB Richman PS Impact of rapid on-site cytologic evaluation during transbronchial needle aspiration Chest 2005 128 869 875 10.1378/chest.128.2.869 16100180

[B10] Herth FJ Eberhardt R Vilmann P Krasnik M Ernst A Real-time endobronchial ultrasound guided transbronchial needle aspiration for sampling mediastinal lymph nodes Thorax 2006 61 795 798 10.1136/thx.2005.047829 16738038 PMC2117082

[B11] Holty JE Kuschner WG Gould MK Accuracy of transbronchial needle aspiration for mediastinal staging of non-small cell lung cancer: a meta-analysis Thorax 2005 60 949 955 10.1136/thx.2005.041525 15994251 PMC1747236

[B12] Gasparini S Silvestri GA Usefulness of transbronchial needle aspiration in evaluating patients with lung cancer Thorax 2005 60 890 891 10.1136/thx.2005.048728 16263944 PMC1747251

[B13] Gould MK Multidisciplinary management of lung cancer N Engl J Med 2004 350 19 10.1056/ENEJMicm030196 15131841

[B14] Fernández-Villar A Botana MI Leiro V Clinical utility of transbronchial needle aspiration of mediastinal lymph nodes in the diagnosis of sarcoidosis in stages I and II Arch Bronconeumol 2007 43 495 500 17919416 10.1016/s1579-2129(07)60114-1

[B15] Fernández-Villar A Leiro V Blanco M Efficacy and safety of the eXcelon transbronchial aspiration needle in mediastinal lymph node enlargement: a case-control study Respiration 2007 74 208 13 10.1159/000097496 17124381

[B16] Altman DG Bland JM Statistics notes. Units of analysis BMJ 1997 314 1874 9224131 10.1136/bmj.314.7098.1874 PMC2127005

[B17] Patel NM Pohlman A Husain A Noth I Hall JB Kress JP Conventional transbronchial needle aspiration decreases the rate of surgical sampling of intrathoracic lymphadenopathy Chest 2007 131 773 778 10.1378/chest.06-1377 17356092

[B18] Martinez-Olondris P Molina-Molina M Xaubet A Transbronchial needle aspiration in the study of mediastinal lymph nodes: yield and cost-effectiveness Arch Bronconeumol 2008 44 290 294 18559217

[B19] Toloza EM Harpole L Detterbeck F McCrory DC Invasive staging of non-small cell lung cancer. A review of the current evidence Chest 2003 123 157S 166S 10.1378/chest.123.1_suppl.157S 12527575

[B20] Baram D Comparison of the diagnostic accuracy of transbronchial needle aspiration for bronchogenic carcinoma and other malignances J Bronchol 2004 11 87 91 10.1097/00128594-200404000-00004

[B21] Varela Lema L Fernández-Villar A Ruano-Ravina A Effectiveness and safety of real time endobronchial ultrasound-guided transbronchial needle aspiration: a systematic review Eur Respir J 2009 33 1156 1164 10.1183/09031936.00097908 19407050

[B22] Herth FJ Annema JT Eberhardt R Endobronchial ultrasound with transbronchial needle aspiration for restaging the mediastinum in lung cancer J Clin Oncol 2008 26 3346 350 10.1200/JCO.2007.14.9229 18519953

[B23] Oki M Saka H Kitagawa C Tanaka S Real-time endobronchial ultrasound-guided transbronchial needle aspiration is useful for diagnosing sarcoidosis Respirology 2007 12 863 868 10.1111/j.1440-1843.2007.01145.x 17986115

[B24] Wong M Yasufuku K Nakajima T Endobronchial ultrasound: new insight for the diagnosis of sarcoidosis Eur Respir 2007 29 1182 1186 10.1183/09031936.00028706 17331972

[B25] Baker JJ Solanki PH Schenk DA Van Pelt C Ramzy I Transbronchial fine needle aspiration of the mediastinum. Importance of lymphocytes as an indicator of specimen adequacy Acta Cytol 1990 34 517 523 2375220

[B26] Trisollini R Tinelli C Cancelleri A Transbronchial leedle aspiration in sarcoidosis: Yield and predictors of a positive aspirate J Thorac Cardiovasc Surg 2008 135 837 842 10.1016/j.jtcvs.2007.11.011 18374764

